# Regulation by FSH of the dynamic expression of *retinol-binding protein 4* in the mouse ovary

**DOI:** 10.1186/s12958-018-0348-8

**Published:** 2018-03-20

**Authors:** Yanwen Jiang, Yun Zhao, Shuxiong Chen, Lu Chen, Chunjin Li, Xu Zhou

**Affiliations:** College of Animal Science, Jilin University, 5333 Xian Road, Changchun, Jilin, 130062 China

**Keywords:** Follicle-stimulating hormone, Retinol, Ovary, Granulosa cells

## Abstract

**Background:**

Ovarian retinoid homeostasis plays an important role in the physiological function of the ovary. Retinol-binding protein 4 (RBP4) acts as the mediator for the systemic and intercellular transport of retinol and is heavily involved in cellular retinol influx, efflux, and exchange. However, the expression patterns and regulatory mechanisms of *Rbp4* in the ovary remain unclear.

**Methods:**

The expression pattern of ovarian *Rbp4* was examined in immature mice during different developmental stages and in adult mice during different stages of the estrous cycle. The potential regulation and mechanisms of ovarian *Rbp4* expression by estrogen and related gonadotropins in mouse ovaries were also investigated.

**Results:**

The present study demonstrated that the ovarian expression of *Rbp4* remained constant before puberty and increased significantly in the peripubertal period. In adult female mice, the expression of *Rbp4* increased at proestrus and peaked at estrus at both the mRNA and protein levels. The protein distribution of RBP4 was mainly localized in the granulosa cell and theca cell layer in follicles. In addition, the expression of *Rbp4* was significantly induced by follicle-stimulating hormone (FSH) or FSH + luteinizing hormone (LH) in combination in immature mouse (3 weeks old) ovaries in vivo and in granulosa cells cultured in vitro, both at the mRNA and protein levels. In contrast, treatment with LH or 17β-estradiol did not exhibit any observable effects on ovarian *Rbp4* expression. Transcription factors high-mobility group AT-hook 1 (HMGA1), steroidogenic factor 1 (SF-1), and liver receptor homolog 1 (LRH-1) (which have been previously shown to be involved in activation of *Rbp4* transcription), also responded to FSH stimulation. In addition, H-89, an inhibitor of protein kinase A (PKA), and the depletion of HMGA1, SF-1, and LRH-1 by small interfering RNAs (siRNAs), resulted in a dramatic loss of the induction of *Rbp4* expression by FSH at both the mRNA and protein levels.

**Conclusions:**

These data indicate that the dynamic expression of *Rbp4* is mainly regulated by FSH through the cAMP-PKA pathway, involving transcriptional factors HMGA1, SF-1, and LRH-1, in the mouse ovary during different stages of development and the estrous cycle.

## Summary sentence

Ovarian *Rbp4* expression remains constant before puberty, increases significantly around puberty in immature mice, and peaks at estrus in adult mice, which is mainly regulated by FSH through the cAMP-PKA pathway partly and involves transcriptional factors HMGA1, SF-1, and LRH-1.

## Background

Retinol (vitamin A) and its derivatives, collectively known as retinoids, play crucial roles in ovarian development and normal physiological function [1]. Retinol is not biologically active per se, and within cells can be oxidized to retinal and retinoic acid (RA) by dehydrogenases. Most of the cellular actions of retinoids can be accounted for by the transcriptional regulatory activity of RA through their nuclear receptors, known as RA receptors (RARs) and retinoid X receptors (RXRs), which associate with RA response elements (RAREs) within the promoters of retinoid-responsive genes [1]. RA in ovarian antral follicles enhanced FSH-mediated ovarian follicular cell differentiation and female fertility, and vitamin A deficiency inhibited oocyte development and decreased ovulated oocytes in mice [2, 3]. RA also plays a crucial role in both nuclear and cytoplasmic maturation of mouse and bovine oocytes [4, 5] and can also stimulate steroidogenesis, such as testosterone production in human theca cells and estradiol production in mouse granulosa cells [1, 6]. In addition, ovarian retinoid levels vary with the estrous cycle [7], and the concentration of retinol is greater in the follicular fluids of the dominant follicles than that of small follicles [8, 9]. However, the regulatory mechanisms of ovarian retinoid homeostasis have not yet been fully understood.

The data from our laboratory suggest that FSH enhances retinol uptake, accumulation, and metabolism in the mouse ovary (unpublished data), but the mechanisms remain unclear. Retinol-binding protein 4 (RBP4), which acts as the mediator for the systemic and intercellular transport of retinol, plays an important role in cellular retinol influx, efflux, and exchange [10]; and seems to play an important role in retinol intercellular transport and accumulation in follicular fluids of the dominant follicles. Evidence shows that the RBP4 immunostaining was observed in the layers of theca and granulosa cells of antral follicles with the most intense staining noted in the cells of large and healthy follicles. Furthermore, the levels of RBP4 and retinol in the fluids of large follicles were higher than those in the fluids of medium or small follicles [8]. High RBP4 levels are also observed in the serum of women with polycystic ovary syndrome (PCOS) and in the fluids from swine follicular cysts [11, 12]. Based on these data [8–12], the regulation of *Rbp4* expression during follicular development remains an interesting and important point of study and would provide an explanation for the possible mechanisms involved in changing ovarian retinoid levels during follicular development.

The regulatory mechanisms of follicular development and ovarian function are primarily realized through neuroendocrine activities in the hypothalamus–pituitary–ovary (HPO) axial, although early stage occurs independently of the HPO axis. Follicle-stimulating hormone (FSH) or FSH+ luteinizing hormone (LH), which are released by the pituitary gland, principally control follicular development and ovulation by regulating estradiol (E_2_) production and the functions of granulosa and theca cells. FSH and LH exert their actions by activating their membrane receptors (namely FSHR and LHR, G-protein coupled receptors) thereby resulting in an increase in intracellular cyclic AMP (cAMP), a second messenger involved in the transduction of hormonal or growth signals that regulate cell functions such as proliferation, differentiation, and metabolism [13, 14]. Elevated cAMP activates protein kinase A (PKA), which phosphorylates certain transcription factors and regulates downstream gene transcription. However, it is not until puberty (at approximately 4 weeks of age in mouse) that gonadotropin pulsatility is sufficient to stimulate fully-grown mature follicles and induce ovulation.

In the adult female mouse (after 6–8 weeks old), under the influence of reproductive hormones (FSH, LH and E_2_), ovarian activities (follicular growth, ovulation, and corpus luteum formation and regression) alternate periodically during the estrous cycle (averages 4–5 days: proestrus for 9–18 h, estrus for about 12 h, metestrus for 24–48 h, and diestrus for 48–72 h) [15, 16]. During proestrus, some recruited follicles in the ovary start to grow rapidly under the stimulation of FSH [17]. During the estrus, the dominant follicles mature and ovulate under the influence of FSH, LH, and estradiol [17–19]. After ovulation, the corpus luteum starts to form under the influence of LH and mice enter metestrus [20]; and during diestrus, the mature corpus luteum releases progesterone which inhibits LH and FSH release by the anterior pituitary and inhibits follicular development [17]. Metestrus terminates with the regression of the corpus luteum under the influence of the pulses of prostaglandin (PG_F2α_) released from the uterus [20, 21].

The structural protein high mobility group AT-hook 1 (HMGA1) is important in promoter regulation by uncovering chromatin and facilitating the recruitment of multiple transcription factors [22]. The *cis*-regulatory sequences of the mouse *Rbp4* gene contain several copies of an AT-rich motif, known to be the binding site for HMGA1 proteins. It has also been reported that HMGA1 can bind the *Rbp4* promoter and recruit the transcription factors steroidogenic factor 1 (SF-1) and liver receptor homolog 1 (LRH-1) to form an activation complex and induce *Rbp4* transcription in Hepa1 mouse hepatoma cells [22].

The present study investigated the expression patterns of *Rbp4* during different stages of development and the estrous cycle. We also explored the potential regulatory mechanisms governing ovarian *Rbp4* expression by estrogen and related gonadotropins in mouse ovaries and examined the involvement of HMGA1, LRH-1, and SF-1. The results of this study are expected to provide mechanistic evidence for the fluctuations in ovarian retinoid levels during follicular development and estrous cycle.

## Methods

### Animals

Immature (3 weeks old, the same below) and adult (8 weeks old, the same below) BALB/c mice were obtained from the Medical Department of Jilin University (Changchun, China). The mice were raised in an environment with controlled temperature (22–24 °C) and humidity (60–70%) in a 12 h light/dark cycle and provided with food and water ad libitum. Vaginal smear tests were performed on adult female mice (8 weeks old), and ovaries were collected from the mice at various stages of the estrous cycle. Some of the adult female mice (8 weeks old) were mated with male mice, and the ovaries of newborn female mice were collected at different developmental stages (postnatal 1, 2, 3, 4 and 5 weeks). To avoid the effect of endogenous gonadotrophins and E_2_, we choose immature female mice (3 weeks old) to investigate hormonal effects on ovarian *Rbp4* expression. They were injected intraperitoneally with a single dose of FSH (10 IU/mouse; Ningbo Second Hormone Factory, Ningbo, China), LH (10 IU/mouse; Ningbo Second Hormone Factory, Ningbo, China), or 17β-estradiol (1 μg/mouse/day in corn oil; Sigma, USA) [23]. Mice were anesthetized with isoflurane and sacrificed via cervical dislocation at 24 or 48 h after injection, and ovarian samples used for RNA isolation were rapidly placed in EZNATM RNA safe stabilizer reagent (Omega, USA) and then stored at − 80 °C. All animal studies were conducted in accordance with the protocol approved by the Animal Care and Use Committee of Jilin University.

### Vaginal smear tests

To retrieve ovarian samples from adult mice (8 weeks old) during each of the four stages of the estrous cycle, we performed the vaginal smear tests to identify the specific estrous cycle stages. Briefly, we placed the tip of a plastic pipette filled with 40 μl of phosphate buffered saline after (PBS; Hyclone, USA) into the mouse vagina, and gently flushed the vagina 3–5 times. Vaginal contents (cells and vaginal fluids) from each mouse were collected into a PCR tube and then smeared onto a glass slide. The evaluation of the vaginal smear images was performed under a microscope IX71 system (Olympus, Japan). Proestrus was characterized by the mostly nucleated and some cornified epithelial cells in the smear; estrus by cornified squamous epithelial cells without visible nuclei; metestrus by the predominance of leucocytes and a few nucleated epithelial and/or cornified squamous epithelial cells; and diestrus was characterized by a predominance of leukocytes [24, 25]. The reader is directed to further citations for a full description [24, 25]. Vaginal smear tests were performed at 08:00 and 20:00 each day. When the stages were identified, the mice were sacrificed via cervical dislocation and their ovaries were collected.

### Immunohistochemistry

Intact ovaries collected from immature mice (3 weeks old) that were that were untreated controls or treated with FSH for 48 h were fixed in 4% paraformaldehyde (Boster, Wuhan, China) for 12 h at 4 °C. After washed with PBS and dehydrated, these tissues then were embedded in paraffin wax (Thermo, USA); and paraffin-embedded tissue sections (5 μm) were deparaffinized. After washing with PBS, the sections were treated with 10 mM citric acid buffer (pH 6.0) (Boster, Wuhan, China) for antigen activation for 15 min in boiling water. The sections were washed with PBS and blocked by 5% (*v*/v) BSA in PBS (Boster, Wuhan, China). Sections were incubated overnight at 4 °C with mouse monoclonal antibody specific to RBP4 at a 1:200 dilution in PBS (Proteintech, USA). In order to validate the specificity of RBP4 antibody, 5% nonimmune goat serum [26–29] or PBS [30] was used as negative controls instead of RBP4 antibody. After washed by 0.3% (*v*/v) Triton X-100 in PBS, sections were incubated with goat-anti-mouse second antibody (Boster, Wuhan, China) for 1 h at room temperature, stained using ABC kits, and counterstained with hematoxylin.

### Follicular granulosa cell isolation and culture in vitro

Primary granulosa cells were isolated from immature female mouse (3 weeks old) ovaries, as described previously [31, 32]. The mice were sacrificed via cervical dislocation after being anesthetized, and the follicles were isolated with no. 5 fine needles. Follicles were then treated with trypsin (Hyclone, USA) for 1 h and filtered using a 100-μm filter (Life Technologies, USA). The isolated granulosa cells were cultured in Dulbecco’s Modified Eagle Medium/F12 1:1 (Hyclone, USA) supplemented with 10% fetal bovine serum (Hyclone, USA), 1% insulin–transferrin–selenium (Sigma, USA), and 1% antibiotics (100 IU/ml penicillin and 100 μg/ml streptomycin; Hyclone, USA) at 37 °C in an atmosphere of 5% CO_2_ in air. Twenty-four hours later, non-adherent cells were removed and adherent cells were treated with FSH (100 IU/L), LH (100 IU/L), 8-Br-cAMP (500 μM; Sigma, USA), or H-89 (10 μM; PKA inhibitor; Beyotime, Beijing, China).

### RNA interference

siRNA oligonucleotides specific for mouse *Hmga1*, *Sf-1*, and *Lrh-1* were designed and synthesized by GenePharma (Shanghai, China). The sequences that provided successful knockdown were *Hmga1* siRNA, 5′-GAGTCAGAAAGAGCCCAGT-3; *Sf-1* siRNA, 5′- GCCTCGATGTGAAATTCCT-3′; and *Lrh-1* siRNA, 5′-GCAGAAGAAAGCCCTCATT-3′. The negative control (NC) siRNA was 5′-TTCTCCGAACGTGTCACGT-3′. Cells were transfected at 50–70% confluency with 50 nM of siRNA duplexes using the FuGENE HD transfection reagent (Roche Applied Science, USA) in accordance with the manufacturer’s instructions. FSH was added to the culture medium 24 h after siRNA transfection and cells were treated for an additional 24 h.

### Total RNA extraction and real-time quantitative PCR assay

Total RNA from ovarian tissue and granulosa cells cultured in vitro were extracted using an RNAprep pure Micro Kit (Tiangen, Beijing, China) and reverse transcribed into cDNA using a PrimeScript RT reagent kit (Takara, Japan) according to the manufacturer’s instructions. Real-time PCR was performed on a sequence detection system (Agilent Technologies, USA) using a SYBR Premix Ex TaqII kit (Takara, Japan). The gene-specific primers (forward and reverse, respectively) that we used for real-time quantitative PCR amplification were as follows: *Rbp4* (NM_011255.3), 5′-AGTCAAGGAGAACTTCGACAAGG-3′, 5′-CAGAAAACTCAGCGATGATGTTG-3′; *Hmga1* (NM_016660.2), 5′-GCAGGAAAAGGATGGGACTG-3′, 5′-AGCAGGGCTTCCAGTCCCAG-3′; *Sf-1* (NM_139051.3), 5′-CCAGACCTTTATCTCCATTGTCG-3′, 5′-AGTGTCATCTGGTCAGCCACCT-3′); *Lrh-1* (NM_030676.3), 5′-TCATGCTGCCCAAAGTGGAGA-3′, 5′-TGGTTTTGGACAGTTCGCTT-3′. The expression level of mouse *β-actin* (NM_007393.3), 5′-TCTGGCACCACACCTTCTA-3′, 5′-AGGCATACAGGGACAGCAC-3′, was used as an internal reference. The relative gene expression levels were calculated using the 2^−ΔΔCt^ method. All primers were obtained from Sangon Biotech (Shanghai, China), all experiments were repeated at least three times.

### Western blot analyses

Protein samples were obtained by homogenizing whole ovaries and by lysing granulosa cells in lysate buffer (Beyotime, Beijing, China) with a 10 μg/ml protease and phosphatase inhibitor cocktail (Thermo, USA). Samples were then centrifuged at 13,000 g at 4 °C. Tissue and cell extracts were normalized to the sample protein concentration, as determined by a protein assay kit (Beyotime, Beijing, China). Proteins (40 μg) were separated via sodium dodecyl sulfate polyacrylamide gel (12%) electrophoresis and transferred to polyvinylidene difluoride membranes. The membranes were then blocked in Tris-buffered saline with Tween 20 (TBST) containing 5% non-fat instant milk and incubated overnight at 4 °C with mouse monoclonal antibodies specific to RBP4 or GAPDH (Proteintech, USA); and rabbit monoclonal antibodies specific to HMGA1, SF-1, or LRH-1 (Abcam, USA) each at 1:5000 dilution. After washing with TBST, the membranes were incubated with horseradish peroxidase-conjugated secondary antibody (Proteintech, USA) for 1 h. Enhanced chemiluminescence (ECL) detection was performed using an ECL system according to the specifications of the manufacturer (Beyotime, Beijing, China). Protein levels were normalized to GAPDH and quantified via densitometry using a Tanon gel imaging system (Tanon, Shanghai, China).

### Statistical analyses

The statistical analyses of the data were conducted via one-way ANOVA followed by Tukey’s multiple-range test. Differences were considered significant at *P* < 0.05. All the statistical analyses were performed using SPSS 22.0 for Windows (StatSoft, USA).

## Results

### Variable expression of *Rbp4* in mouse ovaries during the different stages of development and the estrous cycle

**Fig. 1:**
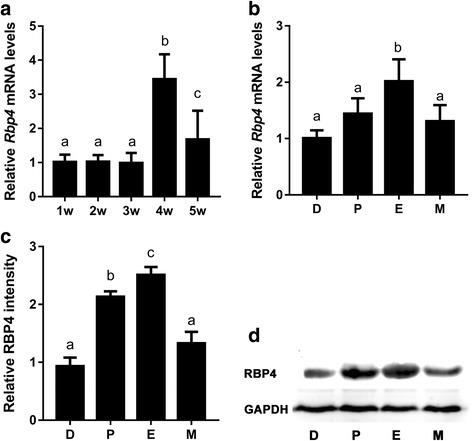
Dynamic expression of *Rbp4* in mouse ovaries during the different stages of development and the estrous cycle. (A) Real-time qPCR analyses of *Rbp4* mRNA levels in mouse ovaries during different developmental stages. Data are presented as means ± SEM, *n* = 8. (B) Relative *Rbp4* mRNA levels in mouse ovaries during diestrus (D), proestrus (P), estrus (E), and metestrus (M). Data are presented as mean ± SEM, n = 8. RBP4 protein levels in the ovaries of mice during the estrous cycle were determined via Western blot analyses (D), and blots were quantified via densitometry (C). The protein levels were normalized to GAPDH. Data are presented as means ± SEM, *n* = 3. Means with different letters represent a significant difference at the 0.05 level

We first performed experiments to investigate the expression pattern of *Rbp4* in whole mouse ovaries during different developmental stages. *Rbp4* mRNA expression in mouse ovaries remained constant at 1 to 3 weeks postnatally and notably increased at 4 weeks (i.e., peripubertally), and dropped at 5 weeks (Fig. 1A). We also investigated the expression patterns of *Rbp4* in whole mouse ovaries during the estrous cycle. The expression of *Rbp4* mRNA in the ovaries of adult mice (8 weeks old) having normal cycles increased at estrus (Fig. 1B), and the levels of RBP4 protein in the ovaries of normally cycling adult mice increased at proestrus and peaked at estrus (Fig. 1C and D). These data imply a potential relationship between *Rbp4* expression and follicular growth [8].

### Effects of gonadotropins and ovarian steroids on *Rbp4* expression in mouse ovaries

**Fig. 2:**
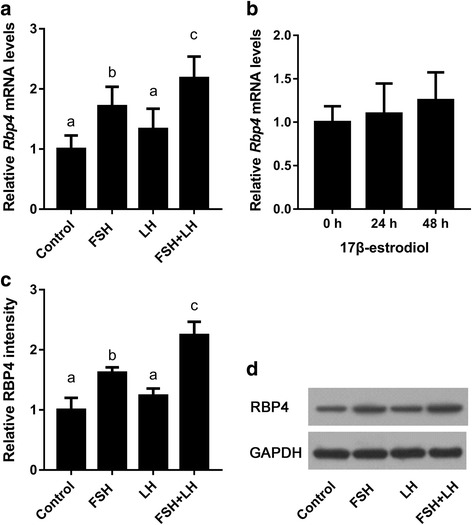
Effects of gonadotropins and ovarian steroids on the expression of *Rbp4* in the 3-week-old mouse ovary. (A) *Rbp4* mRNA expression levels in the ovaries of mice treated with FSH, LH, or FSH + LH for 48 h. Data are presented as means ± SEM, n = 8. (B) *Rbp4* mRNA expression levels in the ovaries of mice treated with 17β-estradiol for 24 or 48 h. Data are presented as mean ± SEM, n = 8. RBP4 protein levels in the ovaries of mice treated with FSH or LH were determined using Western blot analyses (D), and blots were quantified via densitometry (C). The protein levels were normalized to GAPDH. Data are presented as means ± SEM, *n* = 3. Means with different letters represent a significant difference at the 0.05 level

Follicular growth is mainly controlled by gonadotropins and estrogen. Therefore, we determined the effects of these hormones on *Rbp4* expression in mouse ovaries. First, immature mice (3 weeks old) were treated with FSH, LH, or FSH + LH. The results showed that the expression levels of *Rbp4* in whole ovarian samples increased significantly after treatment with FSH (Fig. 2A) or FSH + LH (Fig. 2A), but not with LH alone (Fig. 2A), and the induction with combined FSH + LH was greater than that with FSH alone (Fig. 2A). The levels of RBP4 protein in the ovaries of mice treated with FSH and/or LH in proportion to the mRNA levels (Fig. 2C and D). 17β-Estradiol showed no evident effect on ovarian *Rbp4* expression (Fig. 2B). Thus, *Rbp4* expression in mouse ovaries appeared to be predominantly regulated by FSH.

### Localization of RBP4 protein in mouse ovary

**Fig. 3:**
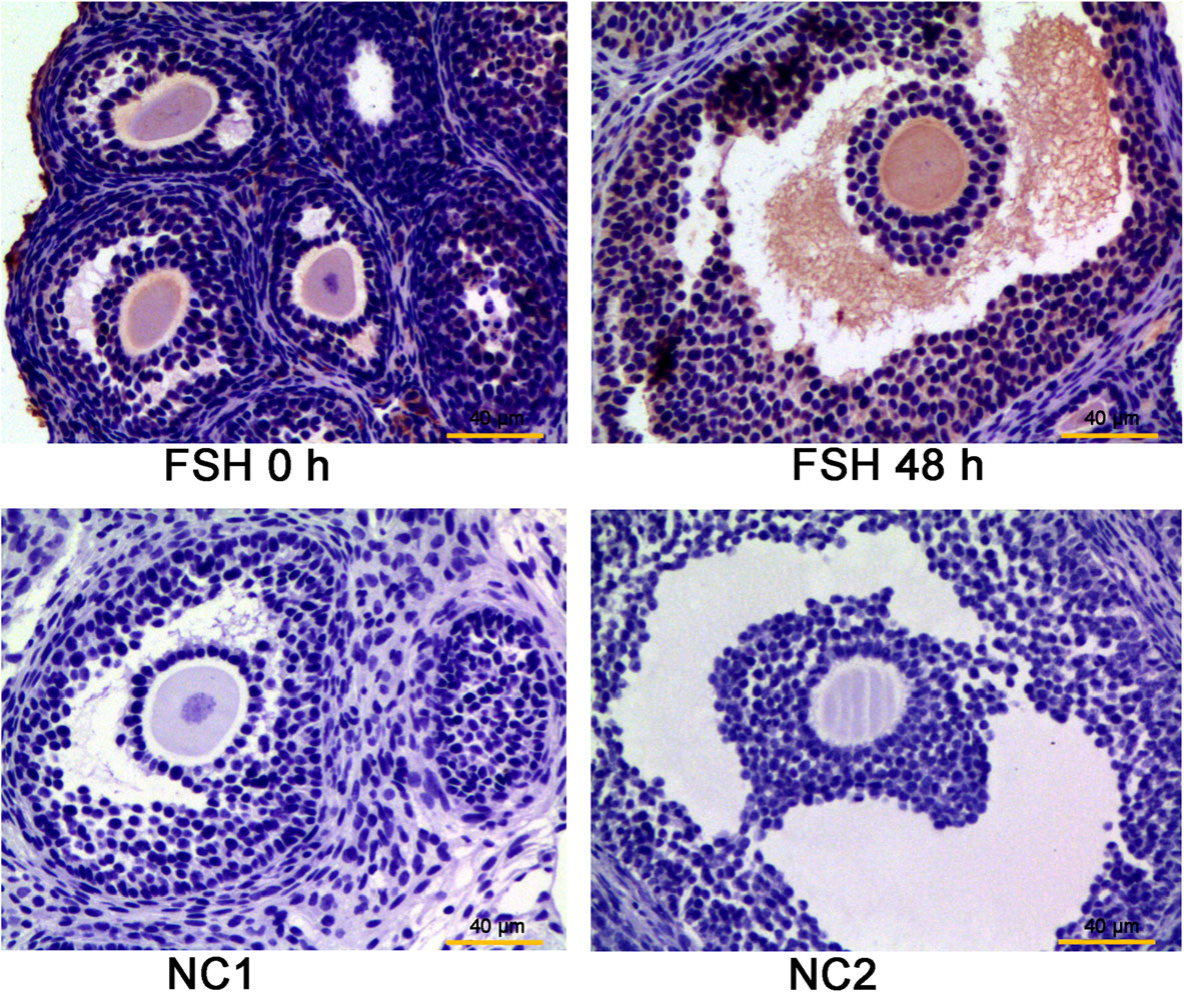
Localization of RBP4 protein in the ovaries of the immature mice (3 weeks of age) treated with or without FSH for 48 h. Negative controls (NC), which were incubated with 5% goat serum (NC1) or PBS (NC2) instead of the primary antibody, were used to validate the specificity of RBP4 antibody. Positive signals for RBP4 were detected in granulosa and theca cell layers. Bar = 40 μm

The localization of RBP4 protein in mouse ovaries was analyzed using immunohistochemical staining with specific antibody (Fig. 3). Positive signals for RBP4 were detected in the layers of granulosa and theca cells, and marked staining was also observed in the follicular antrum of large antral follicles in the ovaries of mice treated with FSH for 48 h (Fig. 3).

### FSH stimulates the expression of *Rbp4* in mouse granulosa cells

**Fig. 4:**
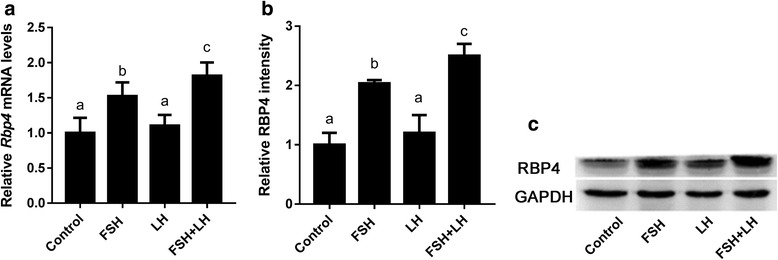
Effects of gonadotropins on the expression of *Rbp4* in mouse granulosa cells. (A) Relative *Rbp4* mRNA levels in mouse granulosa cells treated with FSH and LH for 24 h. Data are presented as means ± SEM, *n* = 4. RBP4 protein levels in mouse granulosa cells treated with FSH and LH for 24 h were determined using Western blot analyses (C), and blots were quantified via densitometry (B). The protein levels were normalized to GAPDH. Data are presented as means ± SEM, *n* = 3. Means with different letters represent a significant difference at the 0.05 level

Granulosa cells are one of the two most important types of gonadotropin-sensitive somatic cells in the mammalian ovary. We examined the effect of FSH and FSH + LH on the expression of *Rbp4* using an in vitro culture model of granulosa cells, and our results showed that the expression of *Rbp4* was induced by both FSH and FSH + LH treatments, and that the induction by combined FSH + LH treatment was greater than that by FSH alone (Fig. 4A, B and C).

### cAMP-PKA pathway mediates FSH-induced *Rbp4* expression

**Fig. 5:**
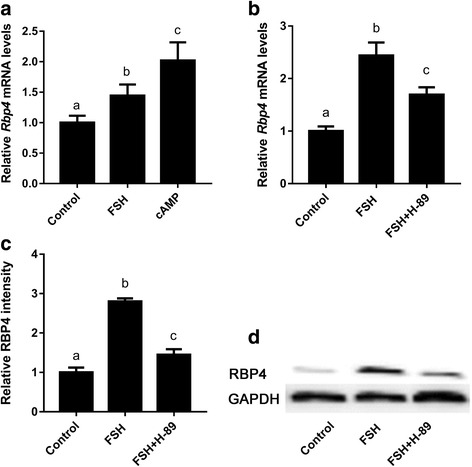
PKA mediates the FSH induction of *Rbp4* in mouse granulosa cells. (A) Activation of PKA by cAMP and FSH increased *Rbp4* mRNA levels. Data are presented as means ± SEM, n = 4. (B) The PKA inhibitor H-89 abrogated FSH induced *Rbp4* mRNA expression. Granulosa cells were pre-incubated with 10 μM H-89 for 1 h, followed by incubation with 100 IU/L FSH for 24 h. Data are presented as means ± SEM, n = 4. (C) Densitometric quantification of Western blots for RBP4 (D). The protein levels were normalized to GAPDH. Data are presented as means ± SEM, n = 3. Means with different letters represent a significant difference at the 0.05 level

As previously reported, FSH and LH perform their actions by activating their membrane receptors, the FSH receptor (FSHR) and the LH receptor (LHR), both of which belong to the G-protein coupled receptors [13, 14]. Stimulation of these receptors results in an increase in intracellular cAMP [13, 14], which promotes the rapid activation of protein kinase A (PKA) [33]. To determine whether the cAMP-PKA pathway was involved in FSH-induced *Rbp4* expression in mouse granulosa cells, we first treated the cells in vitro with 500 μM 8-Br-cAMP (a cAMP agonist) or 10 μM H-89 (a PKA inhibitor). If the cAMP-PKA pathway was involved in FSH induction, 8-Br-cAMP would show similar induction effects and H-89 would prevent FSH induction. As expected, the treatment of mouse granulosa cells with 500 μM 8-Br-cAMP for 24 h resulted in a dramatic increase in *Rbp4* expression (Fig. 5A), and the induction of *Rbp4* by FSH was prevented with 10 μM H-89 (Fig. 5B, C and D). Thus, the cAMP-PKA pathway likely mediates FSH-induced *Rbp4* expression.

### HMGA1, SF-1, and LRH-1 mediate FSH stimulation of *Rbp4*

**Fig. 6:**
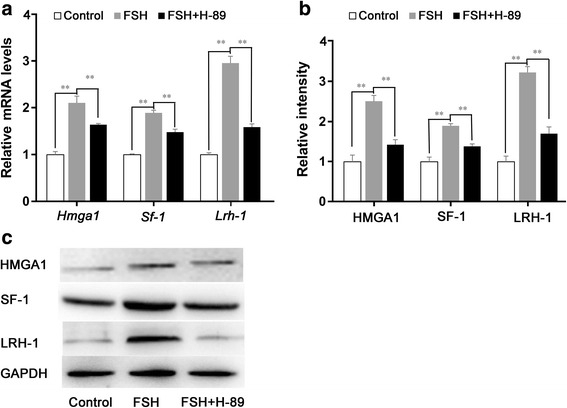
FSH upregulates HMGA1, SF-1, and LRH-1 via PKA. (A) Relative mRNA levels of *Hmga1*, *Sf-1,* and *Lrh-1* in mouse granulosa cells incubated with FSH and H-89 for 24 h. Data are presented as means ± SEM, n = 4. SF-1 and LRH-1 protein levels were determined using Western blot analyses (C), and blots were quantified via densitometry (B). The protein levels were normalized to GAPDH. Data are presented as means ± SEM, n = 3. *p < 0.05, **p < 0.01

**Fig. 7:**
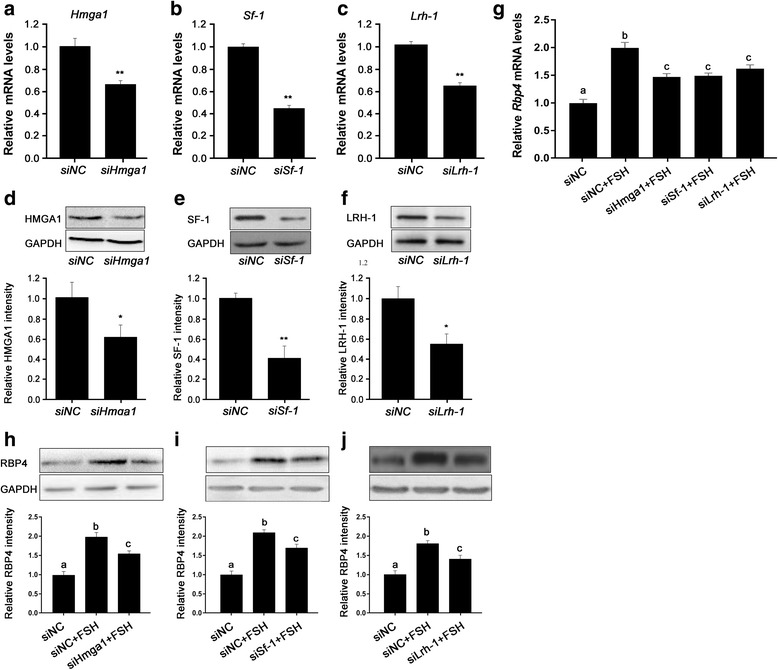
HMGA1, SF-1, and LRH-1 are involved in the FSH induction of *Rbp4* in mouse granulosa cells. mRNA (A) and protein (D) levels of *Hmga1* in mouse granulosa cells transfected with 50 nM *Hmga1* siRNA. The mRNA (B) and protein (E) levels of *Sf-1* in mouse granulosa cells transfected with 50 nM *Sf-1* siRNA. The mRNA (C) and protein (F) levels of *Lrh-1* in mouse granulosa cells transfected with 50 nM *Lrh-1* siRNA. The mRNA levels were determined using real-time PCR. Data are presented as means ± SEM, n = 4. The protein levels were determined using Western blot analyses, and blots were quantified via densitometry. The protein levels were normalized to GAPDH. Data are presented as means ± SEM, n = 3. *p < 0.05, **p < 0.01. (G) The mRNA levels of *Rbp4* in mouse granulosa cells transfected with NC siRNA, *Hmga1* siRNA, *Sf-1* siRNA, or *Lrh-1* siRNA (50 nM). Data are presented as means ± SEM, n = 4. (H), (I), and (J) The RBP4 protein levels in mouse granulosa cells transfected with NC siRNA, *Hmga1* siRNA, *Sf-1* siRNA, or *Lrh-1* siRNA (50 nM). The protein levels were determined using Western blot analyses and blots were quantified via densitometry. The protein levels were normalized to GAPDH. Data are presented as means ± SEM, n = 3. Means with different letters represent a significant difference at the 0.05 level

HMGA1 can bind to the promoter of *Rbp4* and recruit SF-1 and LRH-1 to regulate the transcriptional activity of *Rbp4* [22]. The present study examined the effects of FSH on the expression of these transcription factors, and the results showed that FSH significantly stimulated the expression of these genes in granulosa cells. Moreover, the PKA inhibitor H-89 significantly inhibited FSH induction of these genes (Fig. 6A, B, and C). These data suggest that FSH stimulated the expressions of HMGA1, SF-1, and LRH-1 via PKA. To determine whether HMGA1, SFI, and LRH-1 mediated the FSH induction of *Rbp4*, we sought to knock down the corresponding genes using specific siRNAs. As expected, the expressions of these genes at the mRNA and protein levels were diminished to approximately 50% in cells transfected with 50 nM siRNA compared with controls (Fig. 7A, B, C, D, E, and F). After 24 h of transfection, the cells were treated with FSH for an additional 24 h. The cells were harvested and *Rbp4* mRNA and protein levels were examined. *Rbp4* expression was significantly reduced in cells transfected with *Hmga1* siRNA (Fig. 7G and H); while, knockdown of *Lrh-1* or *Sf-1* also prevented FSH induction of *Rbp4* (Fig. 7G, I, and J). Thus, the transcription factors HMGA1, SF-1, and LRH-1 are likely to be involved in the induction of *Rbp4* expression by FSH*.*

## Discussion

Firstly, the present study presented the dynamic expression patterns for *Rbp4* in mouse ovaries during different stages of development and the estrous cycle. The expression of ovarian *Rbp4* remained constant at 1 to 3 weeks postnatally, notably increased at 4 weeks (i.e., peripubertally), and dropped at 5 weeks. Numerous studies have supported the concept that the gonadotropin-releasing hormone (GnRH) neuronal network generates pulse and surge modes of gonadotropin secretion that are critical for puberty [34], and stimulate the secretion of a mass of local ovarian factors and follicular growth in females. This modulation in *Rbp4* expression may be related to the pituitary gonadotropin wave at the onset of puberty. After puberty, some follicles in ovary start to grow and ovulate irregularly under the influence of serum gonadotropins, and at 5 weeks of age, some mice may be in the follicular-growth phase and some in the post ovulatory period. The different levels of gonadotrophins in these mice may constitute a possible explanation for the drop in *Rbp4* expression at 5 weeks. Furthermore, the expression of *Rbp4* in the ovaries of adult mice (8 weeks old) having normal cycles was found to increase at proestrus and peaked at estrus, the phase with higher serum levels of FSH and LH. In a previous study, higher RBP4 protein concentrations were observed in the follicular fluids of dominant follicles relative to small follicles [8, 9]. The dynamic expression patterns of *Rbp4* in mouse ovaries during different stages of development and the estrous cycle motivated us to pose an alternative hypothesis: that the expression of ovarian RBP4 is affected by gonadotropins and gonadal steroids and is related to follicular growth.

We then investigated the potential regulatory effects of gonadotropins and estrogen on the dynamic expression of *Rbp4* in the mouse ovary. The expression of *Rbp4* was significantly stimulated by administration of FSH and combined FSH + LH, but unaffected by administration of LH or 17β-estradiol. In addition, intense immunostaining was observed in granulosa cell layers of large antral follicles in the ovaries of mice treated with FSH. To determine whether the induction of *Rbp4* by FSH and FSH + LH occurred due to increased expression of *Rbp4* in granulosa cells, we examined primary granulosa cells cultured in vitro. Our results showed that the expression of *Rbp4* was induced by FSH and combined FSH + LH treatments, and that the induction by combined FSH + LH treatment was stronger than that by FSH alone. The administration of LH alone did not exert an effect. Because the primary granulosa cells were isolated from the immature female mouse (3 weeks old) ovaries without FSH or equine chorionic gonadotropin (eCG) pretreatment and with no or low LHR expression, the cells were mainly immature granulosa cells without LHR expression [35]. This may be the reason as to why LH alone manifested no obvious effects on granulosa cells and immature ovaries in the present study. However, LHR in granulosa cells can be induced by FSH [35], and during late maturation of granulosa cells in antral follicles, LH exerts a relatively more notable effect than FSH on cAMP formation [14, 36, 37], suggesting that LHR density was relatively greater than FSHR density or that LHR was more effectively coupled to cAMP generation. These findings may explain the stronger induction of *Rbp4* expression by FSH + LH than by FSH alone, and the higher levels of RBP4 in the follicular fluids of the large antral follicles [8].

To determine whether the induction of *Rbp4* by FSH was mediated by the cAMP-PKA pathway, we examined the effects of the cAMP analog 8-Br-cAMP and the PKA inhibitor H-89 on the induction of *Rbp4* in granulosa cells. The treatment of cells with 500 μM 8-Br-cAMP showed a dramatic increase in *Rbp4* expression, whereas 10 μM H-89 significantly prevented the induction of *Rbp4* by FSH. Thus, the cAMP-PKA pathway likely participates in the induction of *Rbp4* by FSH.

The cis-regulatory sequences of the mouse *Rbp4* gene contain a bipartite promoter, i.e., a proximal region necessary for basal expression and a distal segment that contains several binding sites for the structural HMGA1 proteins that are structural components of chromatin [22, 38]. HMGA1 binds to the adenine thymine (AT)-rich regions of DNA, changes the structure of DNA and recruit transcription factors to the promoter, and facilitates gene transcription [39]. There are several AT-rich motifs homologous to the binding sites of HMGA1 in the promoter region of mouse *Rbp4*, and HMGA1 proteins can bind upstream sequences of the *Rbp4* promoter. In the present study, *Hmga1* was sensitive to FSH and this response was prevented by the PKA inhibitor H-89; whereas the knockdown of *Hmga1* with siRNA resulted in a dramatic loss of FSH-induced *Rbp4* expression. In addition, HMGA1 can interact with SF-1 and LRH-1 and recruit them to the complex [22]. In the present study, the depletion of SF-1 and LRH-1 resulted in a dramatic loss of FSH-induced *Rbp4* expression. SF-1 was shown to be expressed at higher levels in theca cells, and also found in granulosa cells; whereas LRH-1 expression was found to be abundant and highly restricted to the granulosa cells of developing follicles [40, 41]. Consistent with our data, the two transcription factors were shown to be induced in granulosa cells by FSH/cAMP through the activation of PKA [40, 41], as cAMP signals are mediated largely via PKA [33] and can be elevated in granulosa cells by FSH [42, 43] or LH [44]. Thus, the expression of ovarian *Rbp4* was likely regulated by FSH via the cAMP-PKA pathway, involving HMGA1, SF-1, and LRH-1.

## Conclusion

**Fig. 8:**
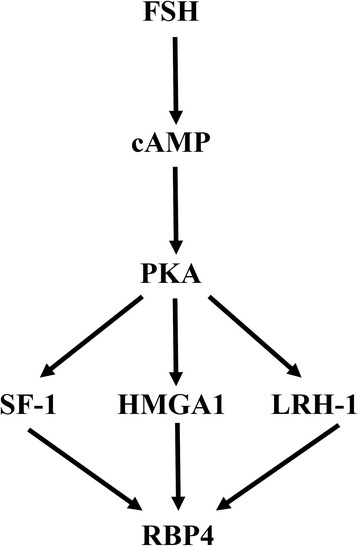
Schematic overview of the proposed pathway involved in the induction of *Rbp4* by FSH. FSH binds to its receptor (FHR) and elevates cAMP levels, which activates PKA. PKA then stimulates the expression of HMGA1, SF-1, and LRH-1, and promotes *Rbp4* transcription

In conclusion, the present study showed a dynamic expression pattern of *Rbp4* in the mouse ovary during different stages of development and the estrous cycle, which was predominantly regulated by FSH. Furthermore, the cAMP-PKA pathway and transcription factors HMGA1, SF-1, and LRH-1 were likely involved in the induction of *Rbp4* by FSH (Fig. 8). This work expands our understanding of the mechanisms underlying the regulation of *Rbp4* expression patterns in the ovary during different stages of development and the estrous cycle. Although further research needs to be conducted in this area, our data are important for the understanding of the molecular regulation of retinol transport and accumulation in the follicular fluids during ovarian follicular growth.

## Abbreviations

8-Br-cAMP: 8-Bromoadenosine 3′:5′-cyclic monophosphate
FSH: Follicle-stimulating hormone
GAPDH: Glyceraldehyde 3-phosphate dehydrogenase
HMGA1: High-mobility group AT-hook 1
LH: Luteinizing hormone
LRH-1: Liver receptor homolog 1
PKA: Protein kinase A
RBP4: Retinol-binding protein 4
SF-1: Steroidogenic factor 1

## References

[1] Jiang YW, Li CJ, Chen L, Wang FG, Zhou X (2017). Potential role of retinoids in ovarian physiology and pathogenesis of polycystic ovary syndrome. Clin Chim Acta.

[2] Kipp JL, Golebiowski A, Rodriguez G, Demczuk M, Kilen SM, Mayo KE (2011). Gene expression profiling reveals Cyp26b1 to be an activin regulated gene involved in ovarian granulosa cell proliferation. Endocrinology.

[3] Kawai T, Yanaka N, Richards JS, Shimada M (2016). De novo-synthesized retinoic acid in ovarian antral follicles enhances FSH-mediated ovarian follicular cell differentiation and female fertility. Endocrinology.

[4] Ikeda S, Kitagawa M, Imai H, Yamada M (2005). The roles of vitamin a for cytoplasmic maturation of bovine oocytes. J Reprod Dev.

[5] Tahaei LS, Eimani H, Yazdi PE, Ebrahimi B, Fathi R (2011). Effects of retinoic acid on maturation of immature mouse oocytes in the presence and absence of a granulosa cell co-culture system. J Assist Reprod Genet.

[6] Wickenheisser JK, Nelson-DeGrave VL, Hendricks KL, Legro RS, Strauss JF, McAllister JM (2005). Retinoids and retinol differentially regulate steroid biosynthesis in ovarian theca cells isolated from normal cycling women and women with polycystic ovary syndrome. J Clin Endocrinol Metab.

[7] Haliloglu S, Baspinar N, Serpek B, Erdem H, Bulut Z (2002). Vitamin a and beta-carotene levels in plasma, corpus luteum and follicular fluid of cyclic and pregnant cattle. Reprod Domest Anim.

[8] Brown JA, Eberhardt DM, Schrick FN, Roberts MP, Godkin JD (2003). Expression of retinol-binding protein and cellular retinol-binding protein in the bovine ovary. Mol Reprod Dev.

[9] Schweigert FJ, Zucker H (1988). Concentrations of vitamin a, beta-carotene and vitamin E in individual bovine follicles of different quality. J Reprod Fertil.

[10] Kawaguchi R, Zhong M, Kassai M, Ter-Stepanian M, Sun H (2015). Vitamin a transport mechanism of the Multitransmembrane cell-surface receptor STRA6. Membranes (Basel).

[11] Jia J, Bai J, Liu Y, Yin JN, Yang P, Yu SQ (2014). Association between retinol-binding protein 4 and polycystic ovary syndrome: a meta-analysis. Endocr J.

[12] Sun YL, Ping ZG, Li CJ, Sun YF, Yi KL, Chen L (2011). Comparative proteomic analysis of follicular fluids from normal and cystic follicles in sows. Reprod Domest Anim.

[13] Richards JS (1994). Hormonal control of gene expression in the ovary. Endocr Rev.

[14] Richards JS, Russell DL, Robker RL, Dajee M, Alliston TN (1998). Molecular mechanisms of ovulation and luteinization. Mol Cell Endocrinol.

[15] McLean AC, Valenzuela N, Fai S, Bennett SA (2012). Performing vaginal lavage, crystal violet staining, and vaginal cytological evaluation for mouse estrous cycle staging identification. J Vis Exp.

[16] Cora MC, Kooistra L, Travlos G (2015). Vaginal cytology of the laboratory rat and mouse: review and criteria for the staging of the estrous cycle using stained vaginal smears. Toxicol Pathol.

[17] Parkening TA, Collins TJ, Smith ER (1982). Plasma and pituitary concentrations of LH, FSH, and prolactin in aging C57BL/6 mice at various times of the estrous cycle. Neurobiol Aging.

[18] Kumar TR, Wang Y, Lu N, Matzuk MM (1997). Follicle stimulating hormone is required for ovarian follicle maturation but not male fertility. Nat Genet.

[19] Montgomery V, Loutradis D, Tulchinsky D, Kiessling A (1988). FSH-induced ovulation in intact and hypophysectomized mice. J Reprod Fertil.

[20] McCracken JA, Custer EE, Lamsa JC (1999). Luteolysis: a neuroendocrine-mediated event. Physiol Rev.

[21] Stocco C, Telleria C, Gibori G (2007). The molecular control of corpus luteum formation, function, and regression. Endocr Rev.

[22] Bianconcini A, Lupo A, Capone S, Quadro L, Monti M, Zurlo D (2009). Transcriptional activity of the murine retinol-binding protein gene is regulated by a multiprotein complex containing HMGA1, p54(nrb)/NonO, protein-associated splicing factor (PSF) and steroidogenic factor 1 (SF1)/liver receptor homologue 1 (LRH-1). Int J Biochem Cell Biol.

[23] Wang C, Dehghani B, Li YX, Kaler LJ, Proctor T, Vandenbark AA (2009). Membrane estrogen receptor regulates experimental autoimmune encephalomyelitis through up-regulation of programmed death 1. J Immunol.

[24] Byers SL, Wiles MV, Dunn SL, Taft RA (2012). Mouse estrous cycle identification tool and images. PLoS One.

[25] Caligioni CS. Assessing reproductive status/stages in mice. Curr Protoc Neurosci. 2009; Appendix 4:Appendix 4I 10.1002/0471142301.nsa04is48 PMC2755182 19575469

[26] Mankhey RW, Bhatti F, Maric C (2005). 17beta-estradiol replacement improves renal function and pathology associated with diabetic nephropathy. Am J Physiol Renal Physiol.

[27] Chen H, Guan R, Lei Y, Chen J, Ge Q, Zhang X (2015). Lymphangiogenesis in gastric cancer regulated through Akt/mTOR-VEGF-C/VEGF-D axis. BMC Cancer.

[28] Mhawech-Fauceglia P, Wang D, Samrao D, Godoy H, Pejovic T, Liu S (2012). Pair-box (PAX8) protein-positive expression is associated with poor disease outcome in women with endometrial cancer. Br J Cancer.

[29] Di Somma S, Marotta M, Salvatore G, Cudemo G, Cuda G, De Vivo F (2000). Changes in myocardial cytoskeletal intermediate filaments and myocyte contractile dysfunction in dilated cardiomyopathy: an in vivo study in humans. Heart.

[30] Ronkainen H, Soini Y, Vaarala MH, Kauppila S, Hirvikoski P (2010). Evaluation of neuroendocrine markers in renal cell carcinoma. Diagn Pathol.

[31] Liang N, Xu YL, Yin YM, Yao GD, Tian H, Wang GS (2011). Steroidogenic Factor-1 is required for TGF-beta 3-mediated 17 beta-estradiol synthesis in mouse ovarian granulosa cells. Endocrinology.

[32] Yao GD, Yin MM, Lian J, Tian H, Liu L, Li X (2010). MicroRNA-224 is involved in transforming growth factor-beta-mediated mouse granulosa cell proliferation and granulosa cell function by targeting Smad4. Mol Endocrinol.

[33] Hunzicker-Dunn M, Maizels ET (2006). FSH signaling pathways in immature granulosa cells that regulate target gene expression: branching out from protein kinase a. Cell Signal.

[34] Herbison AE (2016). Control of puberty onset and fertility by gonadotropin-releasing hormone neurons. Nat Rev Endocrinol.

[35] Camp TA, Rahal JO, Mayo KE (1991). Cellular localization and hormonal regulation of follicle-stimulating hormone and luteinizing hormone receptor messenger RNAs in the rat ovary. Mol Endocrinol.

[36] Yong EL, Baird DT, Hillier SG (1992). Mediation of gonadotrophin-stimulated growth and differentiation of human granulosa cells by adenosine-3′,5′-monophosphate: one molecule, two messages. Clin Endocrinol.

[37] Yong EL, Baird DT, Yates R, Reichert LE, Hillier SG (1992). Hormonal regulation of the growth and steroidogenic function of human granulosa cells. J Clin Endocrinol Metab.

[38] Jessen KA, Satre MA (1998). Induction of mouse retinol binding protein gene expression by cyclic AMP in Hepa 1-6 cells. Arch Biochem Biophys.

[39] Bustin M, Reeves R (1996). High-mobility-group chromosomal proteins: architectural components that facilitate chromatin function. Prog Nucleic Acid Res Mol Biol.

[40] Falender AE, Lanz R, Malenfant D, Belanger L, Richards JS (2003). Differential expression of steroidogenic factor-1 and FTF/LRH-1 in the rodent ovary. Endocrinology.

[41] Hinshelwood MM, Repa JJ, Shelton JM, Richardson JA, Mangelsdorf DJ, Mendelson CR (2003). Expression of LRH-1 and SF-1 in the mouse ovary: localization in different cell types correlates with differing function. Mol Cell Endocrinol.

[42] Mukherjee A, Park-Sarge OK, Mayo KE (1996). Gonadotropins induce rapid phosphorylation of the 3′,5′-cyclic adenosine monophosphate response element binding protein in ovarian granulosa cells. Endocrinology.

[43] Salvador LM, Park Y, Cottom J, Maizels ET, Jones JC, Schillace RV (2001). Follicle-stimulating hormone stimulates protein kinase A-mediated histone H3 phosphorylation and acetylation leading to select gene activation in ovarian granulosa cells. J Biol Chem.

[44] Wang YK, Hao XQ, Yang J, Li J, Zhang MJ (2016). CREB activity is required for luteinizing hormone-induced the expression of EGF-like factors. Mol Reprod Dev.

